# Integrated Transcriptome Analysis Reveals KLK5 and L1CAM Predict Response to Anlotinib in NSCLC at 3rd Line

**DOI:** 10.3389/fonc.2019.00886

**Published:** 2019-09-11

**Authors:** Jun Lu, Qin Shi, Lele Zhang, Jun Wu, Yuqing Lou, Jie Qian, Bo Zhang, Shuyuan Wang, Huimin Wang, Xiaodong Zhao, Baohui Han

**Affiliations:** ^1^Department of Pulmonary Medicine, Shanghai Chest Hospital, Shanghai Jiao Tong University, Shanghai, China; ^2^Department of Oncology, Baoshan Branch of Shuguang Hospital, Shanghai University of Traditional Chinese Medicine, Shanghai, China; ^3^School of Life Science, East China Normal University, Shanghai, China; ^4^Shanghai Center for Systems Biomedicine, Shanghai Jiao Tong University, Shanghai, China

**Keywords:** KLK5, L1CAM, anlotinib, non-small cell lung cancer, transcriptome

## Abstract

The oral multi-targeted tyrosine kinase inhibitor (TKI) anlotinib is effective for non-small cell lung cancer (NSCLC) in clinical trials at 3rd line. However, a fraction of patients remains non-responsive, raising the need of how to identify anlotinib-responsive patients. In the present study, we aimed to screen potential biomarkers for anlotinib-responsive stratification via integrated transcriptome analysis. Comparing with the anlotinib-sensitive lung cancer cell NCI-H1975, we found 1,315 genes were differentially expressed in anlotinib-resistant NCI-H1975 cells. Among the enriched angiogenesis-related genes, we observed high expression of *KLK5* and *L1CAM* was mostly associated with poor clinical outcomes in NSCLC patients through Kaplan-Meier survival analysis in a TCGA cohort. Moreover, an independent validation in a cohort of ALTER0303 (NCT02388919) indicated that high serum levels of KLK5 and L1CAM were also associated with poor anlotinib response in NSCLC patients at 3rd line. Lastly, we demonstrated that knockdown of *KLK5* and *L1CAM* increases anlotinib-induced cytotoxicity in anlotinib-resistant NCI-H1975 cells. Collectively, our study suggested serum levels of KLK5 and L1CAM potentially serve as biomarkers for anlotinib-responsive stratification in NSCLC patients at 3rd line.

## Introduction

Biomarkers play an important role in therapies of non-small cell lung cancer (NSCLC). Genomic features, such as gene amplification, point mutations, gene over-expression, and chromosomal translocation, have been identified as biomarkers in NSCLC (1). NSCLC, as the leading cause of cancer mortality worldwide, has greatly benefited from biomarker investigations. Precision therapies have dramatically improved progression free survival (PFS) and overall survival (OS) of NSCLC patients whose tumors harbor positive driver gene mutations, such as EGFR (19 Del and L858R) (2), rearranged ROS1 (3), or translocated ALK (4). Furthermore, immune checkpoint inhibitors have significantly prolonged PFS and OS in specific advanced NSCLC patients, due to the assessment of PD1/PDL1 expression and tumor mutation burden (TMB) (5–7). Therefore, biomarkers for drug-responsive stratification play crucial roles in NSCLC precision therapy.

Anlotinib is an oral multi-targeted tyrosine kinase receptor inhibitor (TKI) that was recently discovered (8–12). Anlotinib has exhibited its efficacy in various tumor cell line-derived xenograft animal models (9, 11). Clinical trials have revealed that anlotinib is a potent inhibitor for NSCLC therapy at 3rd line (8, 10, 12). Moreover, mechanistic studies indicated that anlotinib is actively involved in anti-angiogenesis and may selectively inhibit VEGFR (2/3), PDGFR (α/β), and FGFR (1–4), and other targets (9–11). Our and other recent studies have revealed some potential biomarkers for anlotinib stratification (13–17). However, due to the complex architecture of angiogenic signaling, the biomarkers for anlotinib-responsive stratification remain further exploration. With the aim of screening potential biomarkers for anlotinib-responsive stratification, in this study we performed integrated transcriptome analysis on anlotinib-resistant NCI-H1975 cells and NSCLC patients both in a TCGA cohort, and examined the stratifying effects in an anlotinib clinical trial cohort (NCT02388919).

## Materials and Methods

### Cell Culture

Human NSCLC cell lines NCI-H1975, PC-9, HCC-827, and A549 were obtained from the ATCC: The Global Bioresource Center (https://www.atcc.org/). All cell lines were validated to exclude mycoplasma contamination using a TransDetect PCR Mycoplasma Detection Kit (TransGen, China). The cells were cultured in RPMI 1640 medium (Gibco, USA) supplement with 10% FBS (Gibco, USA), 0.1 mg/ml streptomycin and 100 U/ml penicillin. All cells were incubated at 37°C and 5% CO_2_ in a humidified incubator.

### Cell Viability Analysis

In total, 1,500 cells per well were cultured in 96-well plates. After incubating with culture medium overnight, the cells were then exposed to anlotinib for 24 h. CCK8 (Dojindo, Japan) was used to evaluate cell viability according to the manufacturer's protocol. The absorbance was measured at 450 nm using a spectrophotometric plate reader (Bio-Tek, USA). Cell viability was performed according to our previous studies (18, 19).

### Establishment of an Anlotinib-Resistant NCI-H1975 Cell Line

**Figure 1:**
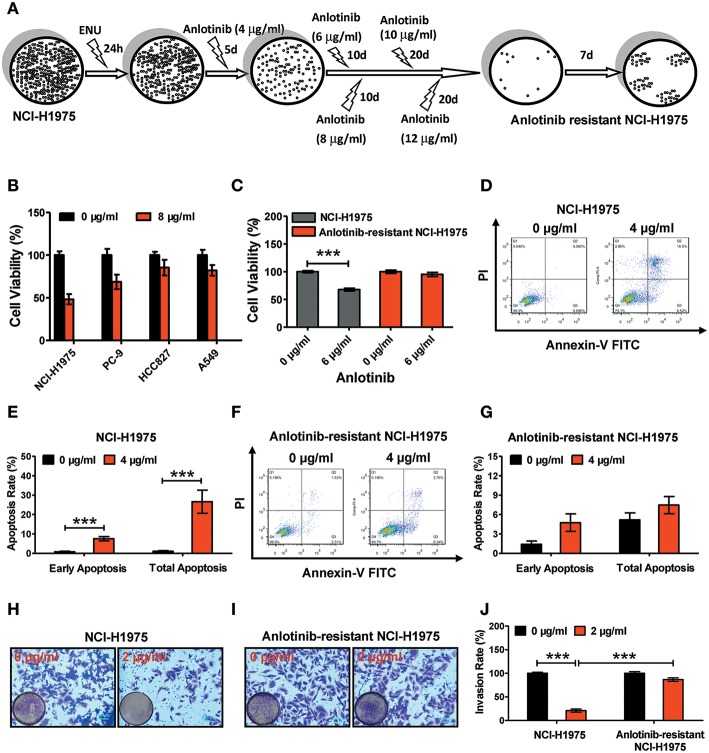
Effect of anlotinib-induced cytotoxicity on NCI-H1975 cells and anlotinib-resistant NCI-H1975 cells. **(A)** Flowchart of anlotinib resistant NCI-H1975 establishment. **(B)** Cell viabilities were assessed using a CCK8 kit after NSCLC cell lines (including NCI-H1975, PC-9, HCC-827, and A549) were exposed to anlotinib (8 μg/ml) for 24 h. Bars = mean ± SD, *n* = 3. **(C)** Cells were seeded in a 96-well plate and then cultured with anlotinib (6 μg/ml) for 24 h. Cell viabilities were measured by CCK8 kit. Bars = mean ± SD, *n* = 3, ***P < 0.001. **(D)** NCI-H1975 cells were treated with anlotinib (4 μg/ml) for 24 h, and then the apoptotic progression was evaluated by flow cytometry. **(E)** Early apoptosis and total apoptosis ratios were analyzed based on the results of flow cytometry. Bars = mean ± SD, *n* = 3, ***P < 0.001. **(F,G)** Anlotinib-resistant NCI-H1975 cells were exposed to anlotinib (4 μg/ml) for 24 h. Apoptotic processes were examined by flow cytometry. Analysis of early apoptosis and total apoptosis were performed based on flow cytometric detection. Bars = mean ± SD, *n* = 3. **(H)** A transwell assay was performed to evaluate NCI-H1975 cell invasion with or without anlotinib (2 μg/ml) for 24 h. **(I)** A transwell assay was performed to evaluate anlotinib-resistant NCI-H1975 cell invasion with or without anlotinib (2 μg/ml) for 24 h. **(J)** Statistical analysis of invasion ratios on NCI-H1975 cells and anlotinib-resistant NCI-H1975 cells. Bars = mean ± SD, *n* = 5, ***P < 0.001.

As our previous study described (20), as shown in Figure 1A, 10^7^ NCI-H1975 cells were exposed to 100 mg/ml ENU (Sigma, USA) for 24 h. Anlotinib administration was performed to screen anlotinib-resistant NCI-H1975 cells. For the first 5 days, NCI-H1975 cells were exposed to anlotinib (4 μg/ml) and the medium was changed every day. Then, anlotinib (6, 8, 10, and 12 μg/ml) treatments were performed over the next two months. The resulting cells (approximately 100 cells) showed viability when exposed to anlotinib (12 μg/ml). After approximately 1 month of culture, the anlotinib-resistant NCI-H1975 cells were used in functional assays.

### Cell Apoptosis Analysis

In total, 5 × 10^5^ cells per well of NCI-H1975 or anlotinib-resistant NCI-H1975 were cultured in six-well plates for 24 h. Then, the cells were exposed to anlotinib for 24 h. To assess the apoptosis rate, an Annexin V-FITC/PI Apoptosis kit (Zoman Biotechnology Co., Ltd, China) was used to determine the phosphatidyl serine and membrane integrity of each cell. Briefly, the anlotinib-treated and anlotinib-untreated cells were stained with annexin V-FITC and PI simultaneously and then detected by flow cytometry (BD LSRFortessa, USA). The ratio of early apoptosis and total apoptosis were analyzed by FlowJo 7.6 (BD, USA).

### Cell Invasion Analysis

Cell invasion was evaluated by transwell assay. One day before the experiment, all cells were incubated in RPMI 1640 (Gibco, USA) for starvation. 5 × 10^4^ NCI-H1975 cells or anlotinib-resistant NCI-H1975 cells per well were then seeded on the top pre-coated chamber in 100 μl RPMI 1640. Five hundred microliter RPMI 1640 containing 15% FBS was added into the lower chamber. After 24 h of incubation, the non-invasive cells were cleaned, and the invasive cells were fixed with 4% PFA for 30 min. The invasive cells were stained with 0.1% crystal violet (Sigma, USA), and photographed using fluorescence microscopy (Nikon, Japan).

### RNA-seq Library

The preparation of RNA-seq library was performed according to our previous studies (18, 21, 22). Briefly, NCI-H1975 cells or anlotinib-resistant NCI-H1975 cells were cultured in 10 cm dishes. Then, 1 ml Trizol reagent (Life Technologies, Inc., USA) was used to lyse the cell samples, followed by total RNA isolation using standard procedures. An Oligotex mRNA Mini Kit (Qiagen, Germany) was used to purify mRNA. Approximately 100 ng mRNA of each sample was used for reverse-transcription, followed by end repair, ligation, using NEBNext Ultra Directional RNA Library Prep Kit (NEB, USA) and PCR amplification (12 cycles) using Q5 High-Fidelity DNA Polymerase (NEB, USA). Lastly, the PCR products were subjected to Illumina sequencing by Next 500 (Illumina, USA). All raw data were deposited at EMBL database under accession number E-MTAB-5997 and E-MTAB-7068.

### Bioinformatics Analysis

Raw sequencing data were mapped to a reference genome (hg38) by Tophat. Cufflinks was used to determine the differential transcription pattern. Kilo-base of per million reads mapped (RPKM) was used to define gene expression level. To screen significant differential genes, we filtered the genes whose gene expression levels were no more than a 2-fold change. Log_2_ (Fold Change) > 1 represented at least 2-fold up-regulation, and log_2_ (Fold Change) < −1 represented at least 2-fold down-regulation. Gene ontology (GO) analysis and Kyoto Encyclopedia of Genes and Genomes (KEGG) pathway analysis were performed using a public bioinformatics resource platform (DAVID, https://david.ncifcrf.gov/) by uploading the differential gene lists.

### Quantitative Real-Time PCR

Total RNA extraction and reverse transcription reactions were performed according to our previous studies (18, 21, 22). Briefly, the mRNA levels of the genes of interest were detected by quantitative real-time PCR (RT-qPCR) using ABI step one plus (Applied Biosystems, USA). GAPDH was used as a control gene for normalization. The relative levels of mRNA were calculated as 2^ΔΔ*Ct*^. All primer sequences used for RT-qPCR are listed in Table S1.

### Transcriptome Analysis of the TCGA Cohort

RNA-seq data and clinical data for NSCLC patients [including lung adenocarcinoma (LUAD) and lung squamous carcinoma (LUSC)] were downloaded from the TCGA portal (https://cancergenome.nih.gov/). The RNA-seq data of normal controls were excluded based on TCGA barcode principle (https://wiki.nci.nih.gov/display/TCGA/TCGA±barcode). After filtering the unqualified samples, 503 LUAD patients and 494 LUSC patients were used for survival analysis. The method of raw data collection was described by the Cancer Genome Atlas Research Network. The correlation analysis of RPKM values and overall survival was performed by R package (survival, version. 2.41-3). Best cutoff value was determined using the “Ward method.” Briefly, to determine the *P*-value, we detected the correlations between each mRNA level and OS. The cutoff value was defined as the lowest *P*-value.

### RNA Interference

RNA interference was performed according to our previous studies (18, 22). NCI-H1975 cells and anlotinib-resistant NCI-H1975 cells were transfected with KLK5 siRNA (5′-GCAUGUUCUCGCCAACAAUTT-3′) or L1CAM siRNA (5′-CAGCAACUUUGCUCAGAGGTT-3′) when they reached 50% confluence, using the Lipofectamine 3000 reagent (Invitrogen, USA). An unrelated, scrambled siRNA was used as a negative control (5′-UUCUCCGAACGUGUCAGGUTT-3′).

### Detection of Serum KLK5 and L1CAM Levels

Twenty-eight peripheral blood samples from patients with refractory advanced NSCLC (time since prior anlotinib treatment: 2 weeks; Registered No. NCT02388919) were provided by Chia-tai Tianqing Pharmaceutical Co Ltd, Jiangsu Province, China. These samples were selected from 294 anlotinib clinical trial participants randomly. The participants received anlotinib as 3rd line or after 3rd line therapy. For each cycle of medication, the patients received anlotinib (12 mg/day) for 2 consecutive weeks and then discontinued for 1 week. Anlotinib treatment was terminated at disease progression or if intolerable toxicity occurred. The patients with stable disease or partial response lasting 80 days were defined as responders while those patients with disease progression ≤ 80 days were defined as non-responders. The patients harboring any driver mutations (detected by standard methods afforded by participant hospitals), such as EGFR, ROS1, and ALK, were defined as positive. All enrolled patients were followed up every 8 weeks until death. The ELISA kit for KLK5 detection was purchased from Abcam. Serum L1CAM levels were determined using the DRG Diagnostics ELISA kit (Marburg, Germany). All experimental procedures were performed according to the manufacturer's protocols.

### Specificity and Sensitively Analysis

For the TCGA cohort of NSCLC patients, the receiver operator characteristic (ROC) curve for predicting OS was generated by the cutoff value of the mRNA level using GraphPad Prism (GraphPad software, version 5, USA). For anlotinib response prediction, the ROC curves for predicting PFS and OS were generated by the cutoff value of the serum protein level.

### Statistical Analysis

There were at least three biological replicates, excluding RNA-seq analysis, for each sample. PFS and OS were summarized as median values and were analyzed using the Kaplan-Meier method. The Mantel-Cox test was used to perform Meier survival analysis in GraphPad Prism 5. Log-rank test, two-tailed Student's *t*-test, or one-way ANOVA with *post-hoc* Bonferroni correction were used to examine the raw data. Differences were considered significant at ^*^P < 0.05, ^**^P < 0.01, and ^***^P < 0.001.

## Results

### Anlotinib-Induced Cytotoxicity Disappeared in Anlotinib-Resistant NCI-H1975 Cells

To verify the anti-tumor effects of anlotinib, we administered anlotinib to NSCLC cell lines (including NCI-H1975, PC-9, HCC827, and A549). After exposure to anlotinib (8 μg/ml) for 24 h, the cell viabilities of those NSCLC cell lines decreased to different degrees (Figure 1B). Among the various lines of NSCLC cells, the NCI-H1975 cells underwent the most cytotoxicity. To investigate the effect of anlotinib resistance, we established anlotinib-resistant NCI-H1975 cells *in vitro* (see methods). We treated NCI-H1975 cells and anlotinib-resistant NCI-H1975 cells with anlotinib simultaneously and then examined the cell viability, cell apoptosis, and cell invasion activity. Under the anlotinib (6 μg/ml) stress, the viability of NCI-H1975 cells decreased remarkably, and the similar phenomenon was not observed in anlotinib-resistant NCI-H1975 cells (Figure 1C). Furthermore, after exposure to anlotinib (4 μg/ml) for 24 h, the apoptosis rate of NCI-H1975 cells increased significantly, while the apoptosis rate of anlotinib-resistant NCI-H1975 cells almost remained unchanged (Figures 1D–G). Consistent with the above results, the invasive ability of anlotinib-resistant NCI-H1975 cells was virtually unaffected, although cells were also exposed to anlotinib (2 μg/ml) for 24 h (Figures 1H–J). These results suggest that anlotinib resistance in NCI-H1975 cells might be attributed to activation/inactivation of tumor survival-related biological processes or signaling pathways.

### Transcriptome Analysis Revealed Anlotinib Resistance in NCI-H1975 Cells Attributed to the Expressions of Angiogenesis-Related Genes

**Figure 2:**
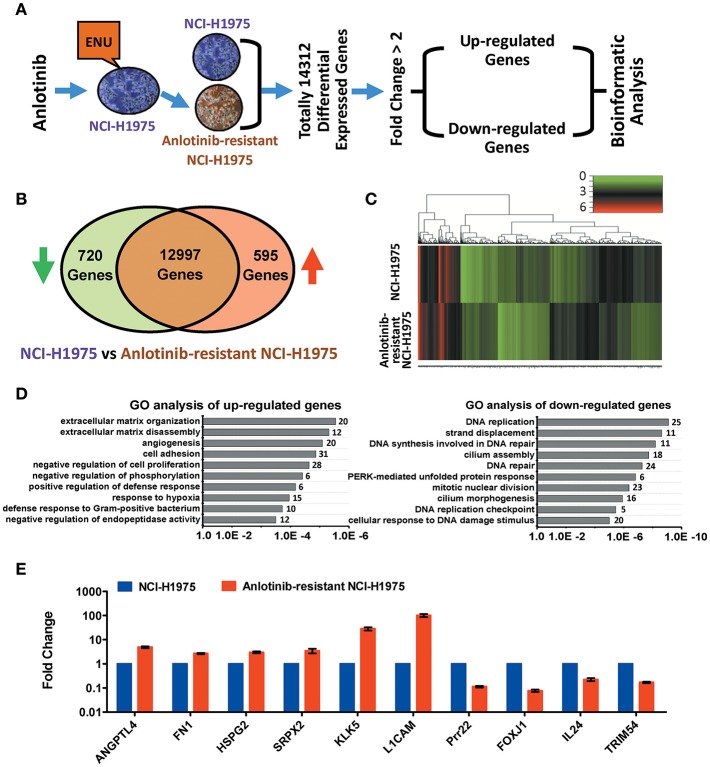
Bioinformatics analysis of up-regulated and down-regulated genes in anlotinib-resistant NCI-H1975 cells. **(A)** Schema of multiple cross-check analysis for screening up-regulated and down-regulated genes in anlotinib-resistant NCI-H1975 cells. **(B)** Venn diagram analysis of differentially expressed genes that were modulated in anlotinib-resistant NCI-H1975 cells. The green arrow represents down-regulation and the red arrow represents up-regulation. **(C)** Heat map representation of differentially expressed genes that were modulated in anlotinib-resistant NCI-H1975 cells. **(D)** GO analysis of up-regulated genes and down-regulated genes in anlotinib-resistant NCI-H1975 cells. Numbers of enriched genes were shown on the right of column. **(E)** mRNA levels of potentially predictive genes detected by RT-qPCR in NCI-H1975 cells and anlotinib-resistant NCI-H1975 cells.

To understand the underlying molecular mechanism of anlotinib resistance in NCI-H1975 cells, we next performed transcriptome profiling analysis on both NCI-H1975 cells and anlotinib-resistant NCI-H1975 cells. The analysis flowchart was shown in Figure 2A. In total, 14,312 differentially expressed genes were found. After excluding inactive genes (fold change ≤ 2), 595 up-regulated genes and 720 down-regulated genes were obtained for subsequent analysis (Figure 2B). Compared with wild type (sensitive cell line), a considerable fraction of genes is differentially expressed in anlotinib-resistant NCI-H1975 cells (Figure 2C). GO and KEGG analysis indicated that the up-regulated genes and down-regulated genes are enriched in multiple biological processes (extracellular matrix organization/disassembly, angiogenesis, cell adhesion, and so on) or signaling pathways (ECM-receptor interaction, antigen processing and presentation, viral carcinogenesis, and so on), suggesting that the modulation of these enriched genes may play an important role in the process of anlotinib resistance (Figure 2D, Table S2). Further analysis suggested that modulation of angiogenesis-related genes (including *ANGPTL4, FN1, HSPG2, SRPX2, KLK5, L1CAM, Prr22, FOXJ1, IL24*, and *TRIM54*) potentially contributes to anlotinib resistance (Figures 2D,E, Tables S3, S4), as anlotinib is a multi-targeted anti-angiogenesis drug for cancer therapy (8–10, 12, 23).

### High mRNA Levels of *KLK5* and *L1CAM* Are Associated With Poor Clinical Outcomes in NSCLC Patients in the TCGA Cohort

**Figure 3:**
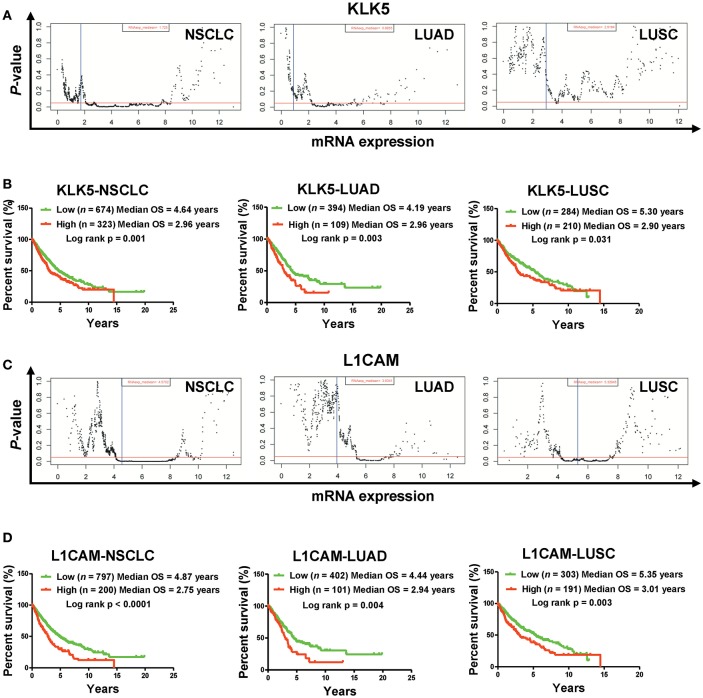
High mRNA levels of *KLK5* and *L1CAM* are associated with poor prognosis in a TCGA cohort. **(A)** The *P*-value examination of the correlations between mRNA expression and clinical outcome in NSCLC patients (including LUAD patients and LUSC patients) in a TCGA cohort. The cutoff value of *KLK5* was determined by the Ward method in NSCLC, LUAD, and LUSC. The red line represents *P* = 0.05. The blue line represents median value. Each dot represents a *P-*value corresponding to mRNA levels. **(B)** The impact of *KLK5* expression on OS in NSCLC patients (including LUAD patients and LUSC patients). NSCLC: *n* = 997, log rank p = 0.003; LUAD: *n* = 503, log rank p = 0.006; LUSC: *n* = 494, log rank p = 0.151. **(C)** The *P*-value of the correlations between mRNA expression and clinical outcome in NSCLC patients (including LUAD patients and LUSC patients). The cutoff value of *L1CAM* was determined by the Ward method in NSCLC, LUAD, and LUSC. **(D)** *L1CAM* expression is associated with OS in NSCLC patients (including LUAD patients and LUSC patients). NSCLC: *n* = 997, log rank *P* < 0.0001; LUAD: *n* = 503, log rank *p* = 0.004; LUSC: *n* = 494, log rank *p* = 0.003.

To understand the clinical significances of the angiogenesis-related genes identified above, we performed survival analysis on NSCLC patients from the TCGA cohort. Kaplan-Meier survival analysis indicated that high mRNA levels of *ANGPTL4, FN1, HSPG2*, and *SRPX2* are associated with poor clinical outcome significantly (Figure S1). However, we also found that down-regulation of *Prr22, FOXJ1, IL24*, and *TRIM54* is also correlated with poor clinical outcome in NSCLC patients (including LUAD and LUSC) (Figure S2). Moreover, our Kaplan-Meier survival analysis showed that high mRNA levels of *KLK5* and *L1CAM* are most significantly associated with poor clinical outcome of NSCLC patients (including LUAD and LUSC) in the TCGA cohort (Figures 2E, 3, Figures S3A–F). Collectively, these results indicated that the activation of *KLK5* and *L1CAM* most likely to result in poor clinical outcome in NSCLC patients and the anlotinib resistance in NCI-H1975 cells.

### Serum Levels of KLK5 and L1CAM Predict Response to Anlotinib in NSCLC Patients

**Figure 4:**
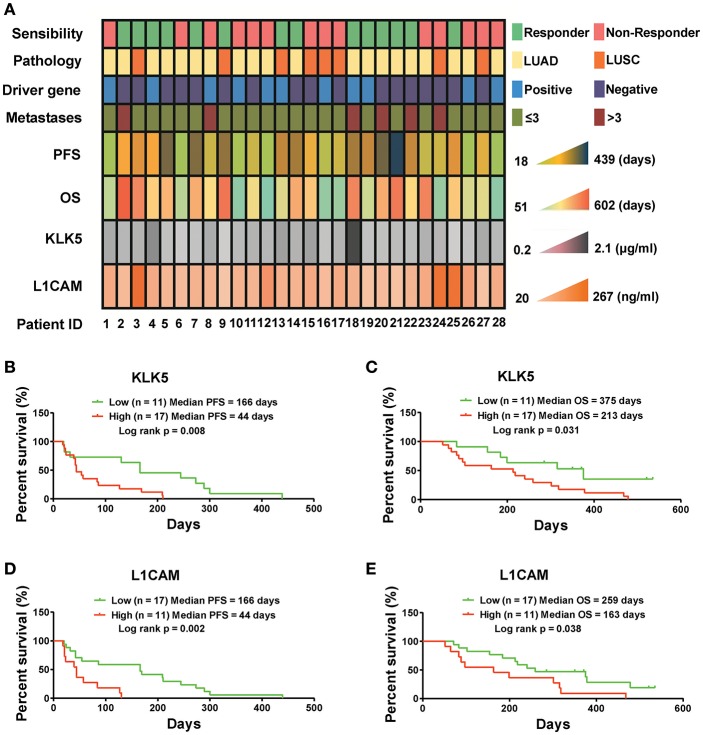
Serum KLK5 and L1CAM levels at baseline are associated with PFS and OS in NSCLC patients treated with anlotinib at 3rd line. **(A)** Serum KLK5 and L1CAM levels correlating to anlotinib response in a 28 patient cohort. Driver gene positive means the patient harboring any one of EGFR, ALK, and ROS1 mutation. Driver gene negative means the patient harboring none of EGFR, ALK, and ROS1 mutation. **(B,C)** Kaplan-Meier curves of PFS and OS via stratifying the serum KLK5 levels in the NSCLC patients treated with anlotinib. *n* = 28, Cutoff-High: 17 patients, Cutoff-Low: 11 patients; PFS: Log rank *p* = 0.008, OS: Log rank *p* = 0.031. **(D,E)** Kaplan-Meier curves of PFS and OS via stratifying the serum L1CAM levels in advanced refractory NSCLC patients treated with anlotinib. *n* = 28, Cutoff-High: 11 patients, Cutoff-Low: 17 patients; PFS: Log rank *p* = 0.002, OS: Log rank *p* = 0.038.

To determine whether serum levels of KLK5 and L1CAM potentially serve as biomarkers for anlotinib-responsive stratification in NSCLC patients at 3rd line, we detected the serum KLK5 and L1CAM levels at baseline in 28 refractory advanced NSCLC patients enrolled in an anlotinib clinical trial (NCT02388919), and then performed response analyses. Previous study has revealed that serum levels of L1CAM could be used as an unfavorable prognostic marker in NSCLC patients (24). However, the implications of KLK5 levels vary in different cancers (25–28). Our raw data including the clinical information and levels of KLK5 and L1CAM were shown in Figure 4A. Further Kaplan-Meier survival analysis suggested that low levels of serum KLK5 in NSCLC patients had a better response to anlotinib than those patients with a high level of serum KLK5 [Low (*n* = 11), Median PFS = 166 days vs. High (*n* = 17), Median PFS = 44 days, *P* = 0.008] (Figure 4B). The NSCLC patients with low levels of serum KLK5 had greater OS benefit from anlotinib treatment [Low (*n* = 11), Median OS = 315 days vs. High (*n* = 17), Median PFS = 240 days, *P* = 0.031] (Figure 4C). Furthermore, Kaplan-Meier survival analysis was performed to examine the predictive value of serum L1CAM level at baseline, and the results indicated the NSCLC patients with low serum L1CAM levels had better PFS and OS [PFS: Low (*n* = 17), Median PFS = 166 days vs. High (*n* = 11), Median PFS = 44 days, *P* = 0.002; OS: Low (*n* = 17), Median OS = 259 days vs. High (*n* = 11), Median OS = 163 days, *P* = 0.038] (Figures 4D,E). The sensitivity and specificity analysis also confirmed that serum KLK5 and L1CAM levels at baseline had preferable predictive value for anlotinib response (Figure S3).

### Knockdown of KLK5 or L1CAM Increases the Sensitivity of NCI-H1975 Cells and Anlotinib-Resistant NCI-H1975 Cells to Anlotinib

**Figure 5:**
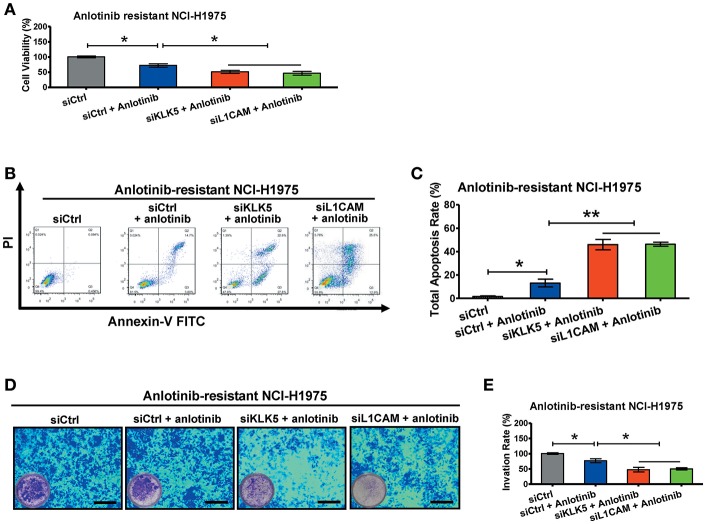
Knockdown of *KLK5* or *L1CAM* contributes to recovery of anlotinib-induced cytotoxicity in anlotinib-resistant NCI-H1975 cells. **(A)** Anlotinib-resistant NCI-H1975 cells were seeded in 96-well plates and then cultured with anlotinib (8 μg/ml), anlotinib + si*KLK5* or anlotinib + si*L1CAM* for 24 h. Cell viabilities were measured by CCK8 kit. Bars = mean ± SD, *n* = 3, *P < 0.05. **(B,C)** Anlotinib-resistant NCI-H1975 cells were treated with anlotinib (8 μg/ml), anlotinib + si*KLK5* or anlotinib + si*L1CAM* for 24 h. The apoptotic progression was evaluated by flow cytometry, and then total apoptosis ratios were analyzed. Bars = mean ± SD, *n* = 3, *P < 0.05, **P < 0.01. **(D,E)** A transwell assay was performed to evaluate and anlotinib-resistant NCI-H1975 cell invasion after exposure to anlotinib (8 μg/ml), anlotinib + si*KLK5* or anlotinib + si*L1CAM* for 24 h. Bars = mean ± SD, *n* = 3, *P < 0.05.

To further investigate the roles of *KLK5* and *L1CAM* in anlotinib resistance, we performed RNA interference assays to evaluate anlotinib-induced cytotoxicity in anlotinib-resistant NCI-H1975 cells. When anlotinib was administered, knockdown of *KLK5* or *L1CAM* significantly decreased the cell viabilities of anlotinib-resistant NCI-H1975 cells (Figure 5A). Meanwhile, anlotinib-induced apoptosis increased significantly, with combined knockdown of *KLK5* or *L1CAM* (Figures 5B,C). Consistent with these results, the invasive ability of anlotinib-resistant NCI-H1975 cells decreased remarkably, after anlotinib administration and knockdown of *KLK5* or *L1CAM* were performed simultaneously (Figures 5D,E). These data indicated that anlotinib-induced cytotoxicity was partially recovered in anlotinib-resistant NCI-H1975 cells after *KLK5* or *L1CAM* knockdown.

## Discussion

Previous studies have demonstrated that anlotinib prolongs PFS and OS in refractory advanced NSCLC patients in clinical trials and indicated that anlotinib may play an important role in anti-angiogenesis and proliferation inhibition (10, 12, 23). These anti-tumor effects may be attributed to anlotinib selectively inhibiting tyrosine kinase receptors, including VEGFR (2/3), PDGFR (α/β), FGFR (1–4), etc. (9–12). Our and other recent studies have been revealed some potential biomarkers for anlotinib stratification (13–15, 17). However, the underlying biomarker for predicting anlotinib-responsive NSCLC patients remain further exploration because of the complex architecture of angiogenic signaling. To address this issue, in this study we sought to screen valuable biomarkers via integrated transcriptome analysis.

Drug resistance is inevitable in the last stage of all anti-tumor drug-related therapeutic regimes (29). Cancer cells can acquire resistance to the anti-tumor drugs by various mechanisms, including over-expression or mutation of the drug target, activation of pro-survival pathways, and eliminative induction of cell death (30). For example, studies have demonstrated the mechanisms of acquired resistance to 1st generation TKIs in NSCLC patients with a positive EGFR mutation, including EGFR^T790M^ mutation, MET amplification, HER-2 mutation, HGF over-expression, etc. (31). In other words, NSCLC patients are not suitable for 1st generation TKI therapy when primary tumors harbor resistant mutations or over-expression. These genomic alterations have been used as biomarkers for anti-tumor drug-responsive stratification (32, 33). Similarly, acquired resistance to anlotinib has been observed in our clinical trials (8, 23). Here, we established anlotinib-resistant NCI-H1975 cells and then demonstrated the resistant effects *in vitro*. Investigation of anlotinib-resistant NCI-H1975 cells may contribute to screening for biomarkers for anlotinib-responsive stratification in NSCLC patients at 3rd line.

Biomarkers play an important role in precision therapy for NSCLC patients. According to gene mutation types, tumor driver gene-derived inhibitors (including EGFR inhibitor, ROS1 inhibitor, and ALK inhibitor) have been screened and used for stratifying treatments in NSCLC patients (2–4). Furthermore, positive PD1/PD-L1 expression and TMB will be used as biomarkers for guiding treatment with immune checkpoint inhibitors in advanced NSCLC patients (5–7). Next generation sequencing (NGS) provided the platform for screening the above biomarkers (6, 7). Our transcriptome analysis suggested that up-regulation of angiogenesis-related genes contributed to anlotinib resistance. Kaplan-Meier survival analysis in the TCGA cohort indicated that the NSCLC patients harboring high mRNA levels of angiogenesis-related genes (including *ANGPTL4, FN1, HSPG2*, and *SRPX2*) have poorer prognosis, suggesting that those patients may be unsuitable for anlotinib therapy.

*KLK5* and *L1CAM* play important roles in cancer progression (including cell proliferation, migration, angiogenesis, invasion, and metastasis) (34, 35), and their expression levels are associated with prognosis. *KLK5* not only regulates *KRT19* expression to increase the malignancy of ovarian cancer cells strongly (36), but also induces miRNA-mediated anti-oncogenic pathways in breast cancer (37). However, *KLK5* plays different roles in different cancers (38, 39). The analysis of correlation between *KLK5* expression and prognosis indicated that higher *KLK5* mRNA level could sever as indicator for predicting unfavorable prognosis in ovarian cancer patients (25, 26) and breast cancer patients (28) and sever as indicator for predicting favorable prognosis in prostate cancer patients (27) and testicular cancer patients (39). *L1CAM* has been characterized as an important pro-angiogenesis molecular via regulating metalloproteinase expression (40). More important, higher serum L1CAM levels have been described as an unfavorable prognostic marker in NSCLC patients (24). Our data indicated that knockdown of *KLK5* or *L1CAM* contributes to increased anlotinib-induced cytotoxicity upon anlotinib-resistant NCI-H1975 cells. Furthermore, our results indicated that up-regulated mRNA levels of *KLK5* and *L1CAM* are simultaneously associated with anlotinib resistance in NCI-H1975 cells and poor prognosis in NSCLC patients. Although the two cohorts (TCGA and ALTER0303) there may be differences in the population profile, but, here we found that low serum levels of KLK5 and L1CAM at baseline are favorable biomarkers for anlotinib-responsive stratification in NSCLC patients (ALTER0303 cohort) at 3rd line.

Collectively, our integrated transcriptome analysis revealed that high mRNA levels of *KLK5* and *L1CAM* are candidate biomarkers for predicting OS in NSCLC patients. High serum KLK5 and L1CAM levels are potentially associated with poor anlotinib response in NSCLC at 3rd line. Knockdown of *KLK5* and *L1CAM* contributes to increasing sensitivity to anlotinib upon anlotinib-resistant NCI-H1975 cells. Collectively, this study suggested serum levels of KLK5 and L1CAM have the potential for clinical application for anlotinib-responsive stratification.

## Data Availability

The datasets generated for this study can be found in the EMBL database under accession number E-MTAB-5997 and E-MTAB-7068.

## Author Contributions

Experiments were conceived and designed by BH, XZ, and JL. Cell assays were performed by JL, QS, BZ, JQ, SW, YL, and LZ. Bioinformatics analysis and statistical analysis were performed by LZ, JW, and JL. The manuscript was written by JL and revised by HW, XZ, and BH.

### Conflict of Interest Statement

The authors declare that the research was conducted in the absence of any commercial or financial relationships that could be construed as a potential conflict of interest.

## References

[1] Kalia M. Biomarkers for personalized oncology: recent advances and future challenges. Metabolism. (2015) 64:S16–21. 10.1016/j.metabol.2014.10.027 25468140

[2] Paez JG Janne PA Lee JC Tracy S Greulich H Gabriel S. EGFR mutations in lung cancer: correlation with clinical response to gefitinib therapy. Science. (2004) 304:1497–500. 10.1126/science.1099314 15118125

[3] Shaw AT Ou SHI Bang YJ Camidge DR Solomon BJ Salgia R. Crizotinib in ROS1-rearranged non-small-cell lung cancer. N Engl J Med. (2014) 371:1963–71. 10.1056/NEJMoa1406766 25264305 PMC4264527

[4] Camidge DR Bang YJ Kwak EL Iafrate AJ Varella-Garcia M Fox SB. Activity and safety of crizotinib in patients with ALK-positive non-small-cell lung cancer: updated results from a phase 1 study. Lancet Oncol. (2012) 13:1011–9. 10.1016/S1470-2045(12)70344-3 22954507 PMC3936578

[5] Herbst RS Baas P Kim DW Felip E Perez-Gracia JL Han JY. Pembrolizumab versus docetaxel for previously treated, PD-L1-positive, advanced non-small-cell lung cancer (KEYNOTE-010): a randomised controlled trial. Lancet. (2016) 387:1540–50. 10.1016/S0140-6736(15)01281-7 26712084

[6] Carbone DP Reck M Paz-Ares L Creelan B Horn L Steins M First-line nivolumab in stage IV or recurrent non-small-cell lung cancer. N Engl J Med. (2017) 376:2415–26. 10.1056/NEJMoa1613493 28636851 PMC6487310

[7] Rizvi NA Hellmann MD Snyder A Kvistborg P Makarov V Havel JJ. Mutational landscape determines sensitivity to PD-1 blockade in non-small cell lung cancer. Science. (2015) 348:124–8. 10.1126/science.aaa1348 25765070 PMC4993154

[8] Han B Li K Zhao Y Li B Cheng Y Zhou J. Anlotinib as a third-line therapy in patients with refractory advanced non-small-cell lung cancer: a multicentre, randomised phase II trial (ALTER0302). Br J Cancer. (2018) 118:654–61. 10.1038/bjc.2017.478 29438373 PMC5846072

[9] Lin B Song X Yang D Bai D Yao Y Lu N. Anlotinib inhibits angiogenesis via suppressing the activation of VEGFR2, PDGFRbeta and FGFR1. Gene. (2018) 654:77–86. 10.1016/j.gene.2018.02.026 29454091

[10] Sun Y Niu W Du F Du C Li S Wang J. Safety, pharmacokinetics, and antitumor properties of anlotinib, an oral multi-target tyrosine kinase inhibitor, in patients with advanced refractory solid tumors. J Hematol Oncol. (2016) 9:105. 10.1186/s13045-016-0332-8 27716285 PMC5051080

[11] Xie C Wan X Quan H Zheng M Fu L Li Y. Preclinical characterization of anlotinib, a highly potent and selective vascular endothelial growth factor receptor-2 inhibitor. Cancer Sci. (2018) 109:1207–19. 10.1111/cas.13536 29446853 PMC5891194

[12] Han B Li K Wang Q Zhang L Shi J Wang Z. Effect of anlotinib as a third-line or further treatment on overall survival of patients with advanced non–small cell lung cancer the ALTER 0303 Phase 3 randomized clinical trial. JAMA Oncol. (2018) 4:1569–75. 10.1001/jamaoncol.2018.3039 30098152 PMC6248083

[13] Lu J Zhong H Chu T Zhang X Li R Sun J Role of anlotinib-induced CCL2 decrease in anti-angiogenesis and response prediction for non-small cell lung cancer therapy. Eur Respir J. (2018) 53:1801562 10.1183/13993003.01562-2018 30578392

[14] Liu Z Wang J Meng Z Wang X Zhang C Qin T CD31-labeled circulating endothelial cells as predictor in anlotinib-treated non-small-cell lung cancer: analysis on ALTER-0303 study. Cancer Med. (2018) 7:3011–21. 10.1002/cam4.1584 PMC6051165 29856135

[15] Wang J Zhao Y Wang Q Zhang L Shi J Wang Z. Prognostic factors of refractory NSCLC patients receiving anlotinib hydrochloride as the third-or further-line treatment. Cancer Biol Med. (2018) 15:443–51. 10.20892/j.issn.2095-3941.2018.0158 30766754 PMC6372914

[16] Charpidou A Kotteas E Gaga M. Towards precision medicine: CCL2, another brick in the wall? Eur Respir J. (2019) 53:1802327. 10.1183/13993003.02327-2018 30846448

[17] Lu J Zhong H Wu J Chu T Zhang L Li H Circulating DNA-based sequencing guided anlotinib therapy in non-small cell lung cancer. Adv Sci. (2019) 7:1900721 10.1002/advs.201900721 PMC6774020 31592412

[18] Lu J Chen J Xu N Wu J Kang Y Shen T. Activation of AIFM2 enhances apoptosis of human lung cancer cells undergoing toxicological stress. Toxicol Lett. (2016) 258:227–36. 10.1016/j.toxlet.2016.07.002 27392435

[19] Lu J Zhang X Shen T Ma C Wu J Kong H. Epigenetic profiling of H3K4Me3 reveals herbal medicine Jinfukang-induced epigenetic alteration is involved in anti-lung cancer activity. Evid Based Complement Alternat Med. (2016). 2016:7276161. 10.1155/2016/7276161 27087825 PMC4818803

[20] Lu J Xu W Qian J Wang S Zhang B Zhang L. Transcriptome profiling analysis reveals that CXCL2 is involved in anlotinib resistance in human lung cancer cells. BMC Med Genomics. (2019) 12(Suppl. 2):38. 10.1186/s12920-019-0482-y 30871526 PMC6416828

[21] Zhang X Wu J Wang J Shen T Li H Lu J. Integrative epigenomic analysis reveals unique epigenetic signatures involved in unipotency of mouse female germline stem cells. Genome Biol. (2016) 17:162. 10.1186/s13059-016-1023-z 27465593 PMC4963954

[22] Lu J Chen J Kang Y Wu J Shi H Fu Y. Jinfukang induces cellular apoptosis through activation of Fas and DR4 in A549 cells. Oncol Lett. (2018) 16: 4343–52. 10.3892/ol.2018.9149 30197670 PMC6126349

[23] Han B Li K Wang Q Zhao Y Zhang L Shi J Efficacy and safety of third-line treatment with anlotinib in patients with refractory advanced non-small-cell lung cancer (ALTER-0303): a randomised, double-blind, placebo-controlled phase 3 study. Lancet Oncol. (2017) 18:S3 10.1016/S1470-2045(17)30759-3

[24] Tischler V Pfeifer M Hausladen S Schirmer U Bonde AK Kristiansen G. L1CAM protein expression is associated with poor prognosis in non-small cell lung cancer. Mol Cancer. (2011) 10:127. 10.1186/1476-4598-10-127 21985405 PMC3198986

[25] Dorn J Magdolen V Gkazepis A Gerte T Harlozinska A Sedlaczek P. Circulating biomarker tissue kallikrein-related peptidase KLK5 impacts ovarian cancer patients' survival. Ann Oncol. (2011) 22:1783–90. 10.1093/annonc/mdq701 21273346

[26] Kim H Scorilas A Katsaros D Yousef GM Massobrio M Fracchioli S. Human kallikrein gene 5 (KLK5) expression is an indicator of poor prognosis in ovarian cancer. Br J Cancer. (2001) 84:643–50. 10.1054/bjoc.2000.1649 11237385 PMC2363783

[27] Yousef GM Scorilas A Chang A Rendl L Diamandis M Jung K. Down-regulation of the human kallikrein gene 5 (KLK5) in prostate cancer tissues. Prostate. (2002) 51:126–32. 10.1002/pros.10067 11948967

[28] Yousef GM Scorilas A Kyriakopoulou LG Rendl L Diamandis M Ponzone R. Human kallikrein gene 5 (KLK5) expression by quantitative PCR: an independent indicator of poor prognosis in breast cancer. Clin Chem. (2002) 48:1241–50. Available online at: http://clinchem.aaccjnls.org/content/48/8/1241 12142380

[29] Holohan C Van Schaeybroeck S Longley DB Johnston PG. Cancer drug resistance: an evolving paradigm. Nat Rev Cancer. (2013) 13:714–26. 10.1038/nrc3599 24060863

[30] Camidge DR Pao W Sequist LV. Acquired resistance to TKIs in solid tumours: learning from lung cancer. Nat Rev Clin Oncol. (2014) 11:473–81. 10.1038/nrclinonc.2014.104 24981256

[31] Yu HA Arcila ME Rekhtman N Sima CS Zakowski MF Pao W. Analysis of tumor specimens at the time of acquired resistance to EGFR-TKI therapy in 155 patients with EGFR-mutant lung cancers. Clin Cancer Res. (2013) 19:2240–7. 10.1158/1078-0432.CCR-12-2246 23470965 PMC3630270

[32] Kim YC Park CK Oh IJ Lim JH Choi YD Cho H Phase II trial of AZD9291 in second line treatment after acquired resistance with T790M mutation detected from circulating tumor DNA (LiquidLung-O-Cohort 2). Ann Oncol. (2017) 28(Suppl. 5):mdx380.062 10.1093/annonc/mdx380.062

[33] Angevin E Spitaleri G Rodon J Dotti K Isambert N Salvagni S. A first-in-human phase I study of SAR125844, a selective MET tyrosine kinase inhibitor, in patients with advanced solid tumours with MET amplification. Eur J Cancer. (2017) 87:131–9. 10.1016/j.ejca.2017.10.016 29145039

[34] Filippou PS Karagiannis GS Musrap N Diamandis EP. Kallikrein-related peptidases (KLKs) and the hallmarks of cancer. Crit Rev Cl Lab Sci. (2016) 53:277–91. 10.3109/10408363.2016.1154643 26886390

[35] Friedli A Fischer E Novak-Hofer I Cohrs S Ballmer-Hofer K Schubiger PA. The soluble form of the cancer-associated L1 cell adhesion molecule is a pro-angiogenic factor. Int J Biochem Cell B. (2009) 41:1572–80. 10.1016/j.biocel.2009.01.006 19401151

[36] Wang P Magdolen V Seidl C Dorn J Drecoll E Kotzsch M. Kallikrein-related peptidases 4, 5, 6 and 7 regulate tumour-associated factors in serous ovarian cancer. Br J Cancer. (2018) 119:823–31. 10.1038/s41416-018-0260-1 30287916 PMC6189062

[37] Sidiropoulos KG White NM Bui A Ding Q Boulos P Pampalakis G. Kallikrein-related peptidase 5 induces miRNA-mediated anti-oncogenic pathways in breast cancer. Oncoscience. (2014) 1:709–24. 10.18632/oncoscience.91 25593998 PMC4278268

[38] Mavridis K Talieri M Scorilas A. KLK5 gene expression is severely upregulated in androgen-independent prostate cancer cells after treatment with the chemotherapeutic agents docetaxel and mitoxantrone. Biol Chem. (2010) 391:467–74. 10.1515/bc.2010.026 20128692

[39] Luo LY Yousef G Diamandis EP. Human tissue kallikreins and testicular cancer. Apmis. (2003) 111:225–33. 10.1034/j.1600-0463.2003.11101261.x 12752266

[40] Na'ara S Amit M Gil Z. L1CAM induces perineural invasion of pancreas cancer cells by upregulation of metalloproteinase expression. Oncogene. (2018) 38:596–608. 10.1038/s41388-018-0458-y 30171263

