# Supplementary material for: Integrated Transcriptome Analysis Reveals KLK5 and L1CAM Predict Response to Anlotinib in NSCLC at 3rd Line
Source: Front Oncol. 2019 Sep 11;9:886. doi: 10.3389/fonc.2019.00886 (PMC6749025; doi:10.3389/fonc.2019.00886)
Supplement: Supplementary file 1 [file Data_Sheet_1.PDF]

Table S1
Primer sequences for RT-qPCR.

Gene	Primer sequences	
	Forward (5'-3')	Reverse (5'-3')
ANGPTL4	CTCTCCGTACCCTTCTCCAC	CGTTGAGGTTGGAATGGCTG
FN1	CGGCCTGGAGTACAATGTCA	GTTGGTGAATCGCAGGTCAG
HSPG2	TGACCAACCCGATGACTTCA	TCGACTAGCTGGAACTCGTG
SRPX2	ACATCATCGAGGAGCTCAGG	GGGGTGACAGGTTCCATGTA
KLK5	CACGAGACGCCTGGTTACTA	TGACACAACAGGAAGCTGGA
L1CAM	CTCCGACAACCACTCAGACT	TCAATCATGCTGTTGGTGGC
Prr22	GACAAGGTTCTGCTGGAGGA	CTGAGTTCCCACCACTGCT
FOXJ1	CCTCCCTACTCGTATGCCAC	CGGATTGAATTCTGCCAGGT
IL24	ATCGTGTCACAACTGCAACC	AGCTGCTTCTACGTCCAACT
TRIM54	CCCACCATTTACAAACGCCA	CTCCGGCTATTGTCCTCGAT
GAPDH	ACCACAGTCCATGCCATCAC	TCCACCACCCTGTTGCT GTA

Table S2

KEGG analysis of differentially expressed genes[a] from NCI-H1975 vs. anlotinib-resistant NCI-H1975.

Category	Item	Pop Hits	P-value
	Up regulated		
hsa04512	ECM-receptor interaction	87	1.22E-04
hsa04612	Antigen processing and presentation	76	1.77E-04
hsa05203	Viral carcinogenesis	205	4.05E-04
hsa05202	Transcriptional misregulation in cancer	168	9.41E-03
hsa04151	PI3K-Akt signaling pathway	345	9.75E-03
hsa04350	TGF-beta signaling pathway	84	2.04E-02
hsa05222	Small cell lung cancer	85	2.17E-02
hsa04145	Phagosome	153	2.96E-02
hsa04115	p53 signaling pathway	67	6.94E-02
hsa05200	Pathways in cancer	393	9.77E-02
	Down regulated		
hsa03008	Ribosome biogenesis in eukaryotes	87	4.88E-04
hsa04110	Cell cycle	124	6.73E-04
hsa03460	Fanconi anemia pathway	53	1.52E-03
hsa03015	mRNA surveillance pathway	91	2.70E-03
hsa04010	MAPK signaling pathway	255	1.90E-02
hsa01130	Biosynthesis of antibiotics	212	4.43E-02
hsa04978	Mineral absorption	46	6.16E-02
hsa04115	p53 signaling pathway	67	6.64E-02
hsa00760	Nicotinate and nicotinamide metabolism	29	6.69E-02
hsa01230	Biosynthesis of amino acids	74	9.26E-02

[a] 595 up-regulated genes (fold change > 2) and 720 down-regulated genes (fold change > 2) are performed KEGG analysis.

Table S3

Angiogenesis-related genes were up-regulated after anlotinib resistant.

Gene	FPKM of NCI-H1975	FPKM of Anlotinib resistant NCI-H1975	log2（fold_change）
ADAM8	5.15142	17.5145	1.765507729
ARHGAP24	2.58327	6.54426	1.341031627
ANPEP	25.9658	88.9526	1.7764241
ANG	2.62262	6.56936	1.324744041
ANGPTL4	57.7981	245.441	2.086282293
COL8A1	7.93869	42.0851	2.406336679
EFNA1	12.0517	26.4613	1.134647275
FN1	76.9231	169.823	1.142543054
HS6ST1	6.2508	17.6688	1.499091313
HSPG2	9.66954	23.8861	1.304652153
LAMA5	4.85326	23.12	2.252115342
MCAM	26.1475	74.0916	1.502636974
MFGE8	19.1493	65.8773	1.782489771
NOV	3.91686	16.3141	2.058349932
PLXND1	2.16936	6.47081	1.576676832
ROBO4	2.50338	11.1389	2.153657563
SAT1	26.6188	65.4577	1.298117376
SRPX2	11.7381	26.196	1.158147632
TSPAN12	5.17147	15.1499	1.550661938
VAV3	1.96358	7.35795	1.905817495

Table S4

Top 10 up-regulated genes and top 10 down-regulated genes in anlotinib-resistant NCI-H1975 cells.

Gene	FPKM of NCI-H1975	FPKM of anlotinib-resistant NCI-H1975	log2（fold_change）
TERC	0	7.45068	-- --
RPL21	0.0569603	125.709	11.10784358
L1CAM	0.909792	138.642	7.251611907
INHBB	0.0711388	6.42422	6.496740851
MGP	0.477045	27.6165	5.855261317
LYPD3	0.918492	41.2678	5.489605564
CYBRD1	0.192746	7.68849	5.317927459
SHH	0.346135	10.7466	4.956401654
KLK5	0.32161	8.5413	4.731071496
IGFBP7	10.1758	218.281	4.422972432
CBLC	9.12578	0.322077	-4.82447033
MT1A	31.3783	1.92576	-4.026267371
EMB	19.8215	1.54888	-3.677768863
PRR22	6.9306	0.560327	-3.628639338
MIR17HG	7.58139	0.678866	-3.481263645
LCN2	8.58003	0.86573	-3.308993633
MAL2	8.92383	0.910054	-3.293638972
FOXJ1	6.11226	0.628339	-3.28209088
IL24	17.9157	2.12069	-3.078618764
TRIM54	6.25618	0.822907	-2.926480722

Supplementary Figure 1

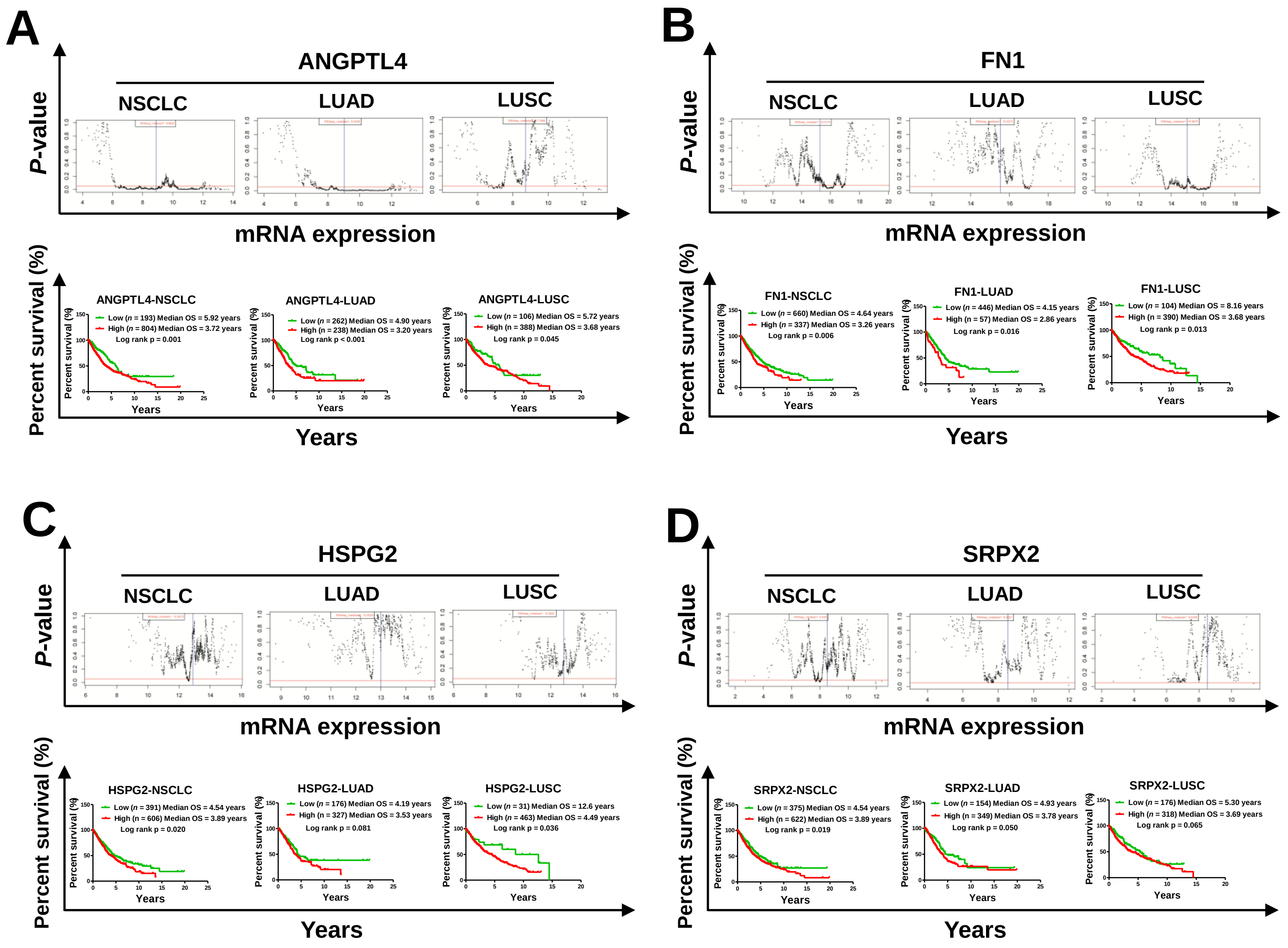

Fig. S1. Correlations between the mRNA levels of angiogenesis-enriched genes and clinical outcomes in NSCLC patients in a TCGA cohort. (A) The *P*-value examination of the correlations between mRNA expressions and clinical outcome of NSCLC (including LUAD and LUSC) patients in a TCGA cohort (n = 997). The cutoff value of *ANGPTL4* was determined by the Ward method. The red line represents *P*-value = 0.05. The blue line represents median value of mRNA levels. Each dot represents a *P* value corresponding to mRNA levels. The impact of *ANGPTL4* expression on OS of NSCLC patients. NSCLC, n = 997, log rank p = 0.013; LUAD, n = 503, log rank p = 0.001; LUSC, n = 494, log rank p = 0.177. (B) *FN1*

expression is associated with OS in NSCLC patients. NSCLC, *n* = 997, log rank p = 0.006; LUAD, *n* = 503, log rank p = 0.166; LUSC, *n* = 494, log rank p = 0.013. The cutoff value of *FN1* was determined by the Ward method. (C) The impact of *HSPG2* expression on OS of NSCLC patients. NSCLC, *n* = 997, log rank p = 0.020; LUAD, *n* = 503, log rank p = 0.086; LUSC, *n* = 494, log rank p = 0.192. The cutoff value of *HSPG2* was determined by the Ward method. (D) *SRPX2* expression is associated with OS in NSCLC patients. NSCLC, *n* = 997, log rank p = 0.033; LUAD, *n* = 503, log rank p = 0.100; LUAD, *n* = 494, log rank p = 0.414. The cutoff value of *SRPX2* was determined by the Ward method.

Supplementary Figure 2

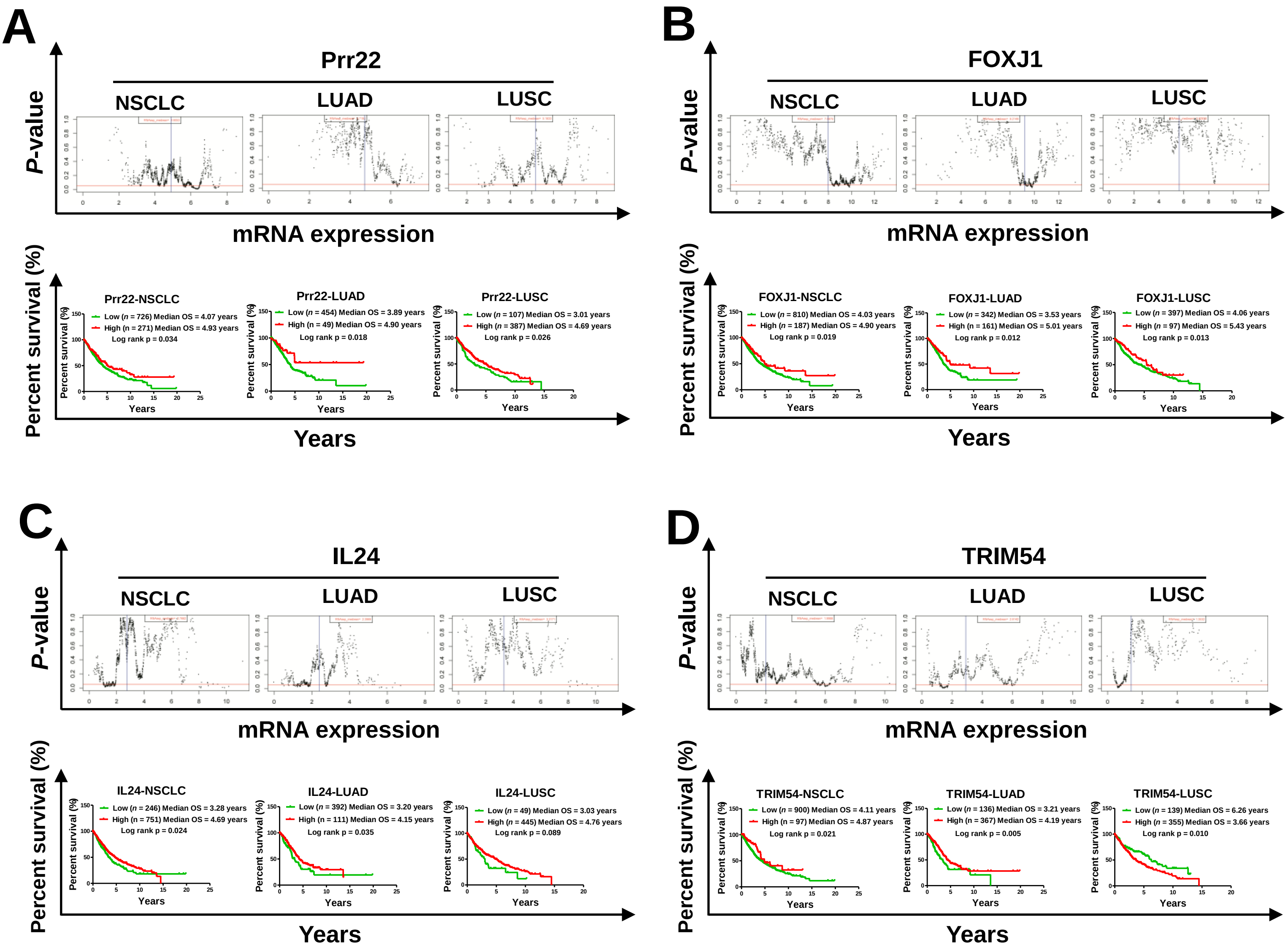

Fig. S2. Correlation analysis between mRNA expression of *Prr22, FOXJ1, IL24* and *TRIM54* and NSCLC outcomes in a TCGA cohort. (A) The *P*-value examination of the correlations between mRNA expressions and clinical outcome of NSCLC (including LUAD and LUSC) patients in a TCGA cohort (n = 997). The cutoff value of *Prr22* was determined by the Ward method. The red line represents *P*-value = 0.05. The blue line represents median value of mRNA levels. Each dot represents a *P* value corresponding to mRNA levels. The impact of *Prr22* expression on OS in NSCLC patients in the TCGA cohort. NSCLC, n = 997, log rank p = 0.034;

LUAD, *n* = 503, log rank p = 0.252; LUSC, *n* = 494, log rank p = 0.026. (B) Correlation analysis of *FOXJ1* expression and overall survival in NSCLC patients in the TCGA cohort. NSCLC: *n* = 997, log rank p = 0.063; LUAD: *n* = 503, log rank p = 0.048; LUSC: *n* = 494, log rank p = 0.013. The cutoff value of *FOXJ1* was determined by the Ward method. (C) The impact of *IL24* expression on overall survival in NSCLC patients in the TCGA cohort. NSCLC: *n* = 997, log rank p = 0.666; LUAD: *n* = 503, log rank p = 0.035; LUSC: *n* = 494, log rank p = 0.773. The cutoff value of *IL24* was determined by the Ward method. (D) Correlation analysis of *TRIM54* expression and overall survival in NSCLC patients in the TCGA cohort. NSCLC: *n* = 997, log rank p = 0.103; LUAD: *n* = 503, log rank p = 0.005; LUSC: *n* = 494, log rank p = 0.085.The cutoff value of *TRIM54* was determined by the Ward method.

Supplementary Figure 3

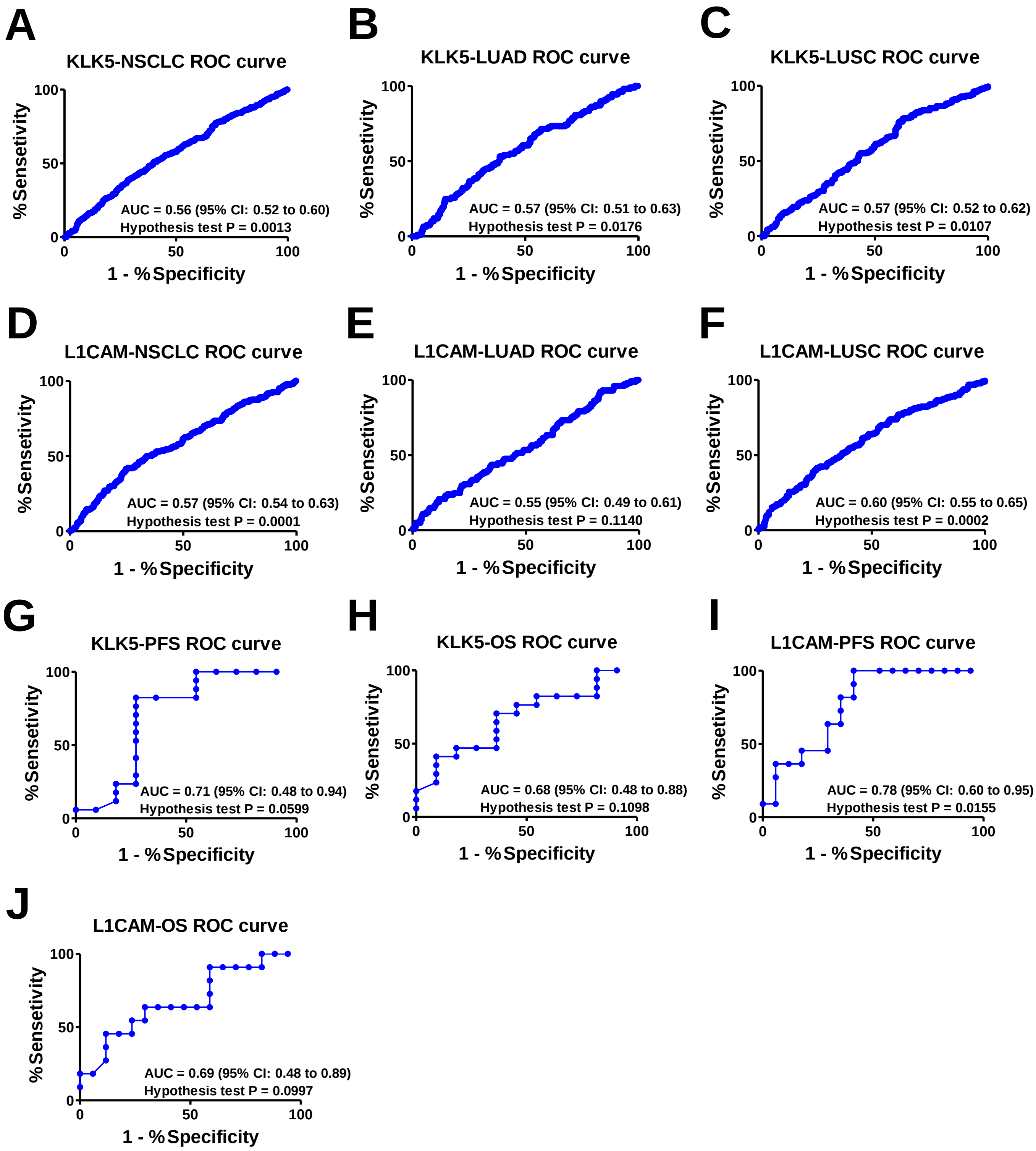

Fig. S3. Sensitivity and specificity analysis of KLK5 and L1CAM in the TCGA cohort and anlotinib clinical trial cohort, respectively. (A-F) ROC curve for the

correlation of *KLK5* and *L1CAM* mRNA levels with OS in the TCGA NSCLC cohort, TCGA LUAD cohort and TCGA LUSC cohort, respectively. (G-J) ROC curve analysis of plasma KLK5 and L1CAM levels linked with PFS and OS in the anlotinib clinical trial cohort.
